# Mind the Academic Gap: Industry Support in the United States for Pediatric Versus Adult Rheumatologist–Authors

**DOI:** 10.1002/acr2.90170

**Published:** 2026-11-04

**Authors:** William Stohl, Krishan Parikh, Anuj Parikh, Sheldon M. Stohl

**Affiliations:** ^1^ Division of Rheumatology, Department of Medicine University of Southern California Keck School of Medicine Los Angeles; ^2^ University of Southern California Los Angeles; ^3^ University of California Los Angeles; ^4^ Department of Anesthesiology and Critical Care Hadassah Hebrew University Medical Center Jerusalem Israel; ^5^ Department of Anesthesiology and Critical Care Children's Hospital Los Angeles Los Angeles California

## Abstract

**Objective:**

Cognitive pediatric subspecialties, including rheumatology, struggle more than their adult counterparts to recruit young physicians. Commercial industry often subsidizes biomedical research and supplements physician income. Reasoning that availability and extent of extramural financial support may steer budding academically oriented practitioners toward or away from particular disciplines, we used published authors as surrogates for academic physicians and compared industry support to pediatric rheumatologist–authors (PRAs) and adult rheumatologist–authors (ARAs).

**Methods:**

We vetted authors of publications in the American College of Rheumatology journals in calendar years 2023 to 2024 for US affiliation, subspecialty, sex, *h*‐index (measure of author impact), and receipt of industry payments during the 2017 to 2023 interval. Differences between PRAs and ARAs in frequency and magnitude of research‐associated and nonresearch‐associated payments were determined. Associations among industry payments, author sex, and *h*‐index were also determined.

**Results:**

A total of 152 of 199 US PRAs (76.4%) and 567 of 710 US ARAs (79.9%) received industry payments (*P* = 0.286). Median total, nonresearch‐associated, and research‐associated payments to PRAs were 27% ($5,744 vs $21,030), 5% ($244 vs $4,905), and 0% ($0 vs $270) of those to ARAs (*P* < 0.001, *P* = 0.018, *P* < 0.001, respectively). These differences cannot be explained by differences in sex distribution or author impact. Among recipients of research‐associated payments, nonresearch‐associated payments were considerably lower to PRAs than to ARAs at any level of research‐associated payment.

**Conclusion:**

Although both US PRAs and ARAs commonly receive industry payments, the magnitude of payment per recipient is far lower to PRAs than to ARAs. We appeal to industry and to the rheumatology community to boost support of academic pediatric rheumatology.

## INTRODUCTION

Physician shortage in the United States is an ongoing concern and is predicted to steadily increase through at least 2030.^1^ Insufficient entry of recruits into medicine as well as premature exit of practicing physicians from medicine are both due, in part, to real or perceived inadequate compensation. Physician shortages are not distributed evenly across medical specialties, with some disciplines more severely affected than others. Cognitive specialties struggle more than procedural specialties to recruit young physicians to fellowship and attending positions, and, among cognitive disciplines, pediatric subspecialties struggle more than do their adult counterparts.^2^ Pediatric rheumatology, like several other pediatric subspecialties, is a discipline that is “feeling the pain.” Although rheumatologists are often viewed as a monolithic entity, important workforce differences exist between US pediatric rheumatologists (PRs) and US adult rheumatologists (ARs). Whereas the number of applicants to adult rheumatology training programs consistently exceeds the number of available positions,^3^ substantial numbers of positions in pediatric rheumatology training programs consistently remain unfilled.^4^ Factors contributing to insufficient interest in pediatric rheumatology include pedagogic ones, such as meager exposure of pediatric residents to the field,^4^ and monetary ones, with compensation to PRs lagging behind that to general pediatricians.^5^ These deterrents have contributed, and likely will continue to contribute, to shortages of PRs nationwide through at least 2040.^6^ Meanwhile, the unmet need of children with pediatric rheumatic diseases persists.

US academic programs are the major sources of future US physicians, and US academic rheumatology programs are no exception. These training programs are heavily dependent on financial support from government, foundations, philanthropic donors, and industry. With uncertainties surrounding the stability of US federal funding,^7^ the role of nonfederal funding, including that from industry, assumes increasing importance.

Support from industry can have two beneficial effects on academic rheumatology programs. First, research‐associated industry payments can provide resources for meaningful investigations conducted by trainees and faculty that help sustain the scholarly atmosphere so vital to enticing (and retaining) gifted residents to careers in rheumatology. Second and notwithstanding the concerns of potential conflicts of interest,^8^, ^9^ nonresearch‐associated industry payments can supplement the notoriously low salaries of academic rheumatologists, helping to keep talented educators and investigators in the academic fold and permitting them to train the PRs and ARs of the future.

Previous studies using public data available at the Centers for Medicare and Medicaid Services Open Payments database have documented that US rheumatologist–authors are frequent recipients of industry payments.^10^, ^11^ Of note, these studies lumped all rheumatologist–authors as a single group and did not distinguish between PR‐authors (PRAs) and AR‐authors (ARAs). We reasoned that industry payments to PRAs versus those to ARAs would serve as a barometer of the support that industry gives to pediatric versus AR academicians. Accordingly, we turned to the three American College of Rheumatology (ACR) journals, *Arthritis & Rheumatology*, *Arthritis Care & Research*, and *ACR Open Rheumatology*, which collectively cover the full gamut of topics of interest to PRs and ARs. We vetted the publications in the January 2023 through December 2024 issues of each of these journals and found that both research‐associated and nonresearch‐associated industry payments to US PRAs were markedly lower than those to US ARAs. These differences cannot be explained by differences between the two groups in sex distribution or author impact, and they sound a cry for assistance from industry and the broader rheumatology community on behalf of PRAs and academic pediatric rheumatology.

## MATERIALS AND METHODS

### Institutional review board approval

All data collected were obtained from public sources, so no institutional review board approval was needed.

### Vetted journals

The January 2023 through December 2024 issues of *Arthritis & Rheumatology*, *Arthritis Care & Research*, and *ACR Open Rheumatology* (24 issues each) were vetted for publications characterized as brief reports, classification criteria, commentaries, concise communications, editorials, full‐length reports, guidelines, journal club, notes from the field, original articles, perspectives, reviews, summit summary, or white paper. Publications that appeared concurrently in more than one ACR journal (eg, guidelines) were vetted as a single publication and assigned to *Arthritis & Rheumatology*.

### Selection of authors

All named authors of the publications were vetted for US affiliations, and US authors were vetted for a National Provider Identifier (NPI) number. Because the sex and taxonomy of each provider with an NPI number is listed (https://npiregistry.cms.hhs.gov/search), we could establish the sex of each NPI number–bearing individual and his/her status as a physician.

### OPD

US physician–authors were vetted for payments from industry during the 2017 to 2023 interval. The provider type and provider specialty are listed for each recipient of industry payments. For PRs, the listed provider type is “Physician,” and the listed provider specialty is “Allopathic & Osteopathic Physicians | Pediatrics | Pediatric Rheumatology.” For ARs, the listed provider type is “Physician,” and the listed provider specialty is “Allopathic & Osteopathic Physicians | Internal Medicine | Rheumatology.” For a provider for whom a subspecialty is not listed or who had not received any industry payments during the 2017 to 2023 interval, an internet search was performed to determine whether the provider is listed as a PR, AR, or neither.

Payments to physicians are separated into four categories on OPD: (1) general payments (direct payments to the physician that are not associated with a research study), (2) research payments (direct payments to the physician in the context of a research study), (3) associated research funding (funding for a research study for which the physician is an investigator but the payment does not go directly to the physician), and (4) ownership and investment interest (includes both the actual dollar amount invested and the value of the ownership or investment interest to the physician). For our analyses, categories 1 and 4 constitute “nonresearch‐associated payments” and categories 2 and 3 constitute “research‐associated payments.”

### *h*‐Index

US rheumatologist–authors were vetted through the Web of Science (Clarivate) for *h*‐index, a metric that measures the impact of an author's publications and is defined as the number of publications by that author with citation numbers equal to or greater than *h*.^12^ For example, for an author with 100 publications, the *h*‐index will be 10 if 10 of these publications have received ≥10 citations, 20 if 20 of these publications have received ≥20 citations, and 100 if all 100 have received ≥100 citations. Because an author's *h*‐index can increase over time as he/she publishes more papers and is cited more frequently, the *h*‐indexes of all authors who received industry payments were determined within a single three‐day period.

### Industry payments

For US physician–authors with at least one OPD entry, the physician type and dollar amount for each OPD category were recorded. Differences between PRAs and ARAs in dollars received for research‐associated and nonresearch‐associated payments and in *h*‐indexes were determined. Associations between extent and magnitude of industry payments, author sex, and *h*‐index were determined.

### Statistics

All analyses were performed using SigmaPlot software (Inpixon). For categorical variables, chi‐squared testing was performed unless the expected cell count was five or fewer, in which case Fisher exact test was performed. Results are presented as medians (interquartile range) and as means. Because the data for the continuous variables were not normally distributed, nonparametric testing was performed by the Mann–Whitney rank‐sum test between two groups, and correlations were calculated using Spearman rank order correlation. *P* ≤0.05 was considered significant for all tests.

## RESULTS

### Lower industry payments to US PRAs than to US ARAs

We identified 1,030 publications in the January 2023 through December 2024 issues of *Arthritis & Rheumatology* (401 publications), *Arthritis Care & Research* (463 publications), and *ACR Open Rheumatology* (166 publications), with 4,413 named authors (3,266 unique individuals) having a US affiliation. Of these, 1,480 are physicians, of whom 710 are ARs and 199 are PRs. During the 2017 to 2023 interval, 567 (79.9%) of these ARAs and 152 (76.4%) of these PRAs received industry payments (*P* = 0.286). These similar percentages notwithstanding, nonresearch‐associated, research‐associated, and total payments to the 152 PRAs were significantly lower than those to the 567 ARAs (Figure 1A–C). Moreover, significantly lower payments to the PRAs than to the ARAs were observed for all of the individual OPD payment categories other than ownership (Table 1). The aggregate nonresearch‐associated, research‐associated, and total payments were $2,345,683, $21,868,783, and $24,214,466 (mean $15,432, $143,874, and $159,306 per recipient) to the PRAs compared to $298,369,856, $322,248,055, and $620,617,911 (mean $526,225, $568,339, and $1,094,564 per recipient) to the ARAs.

**Figure 1:**
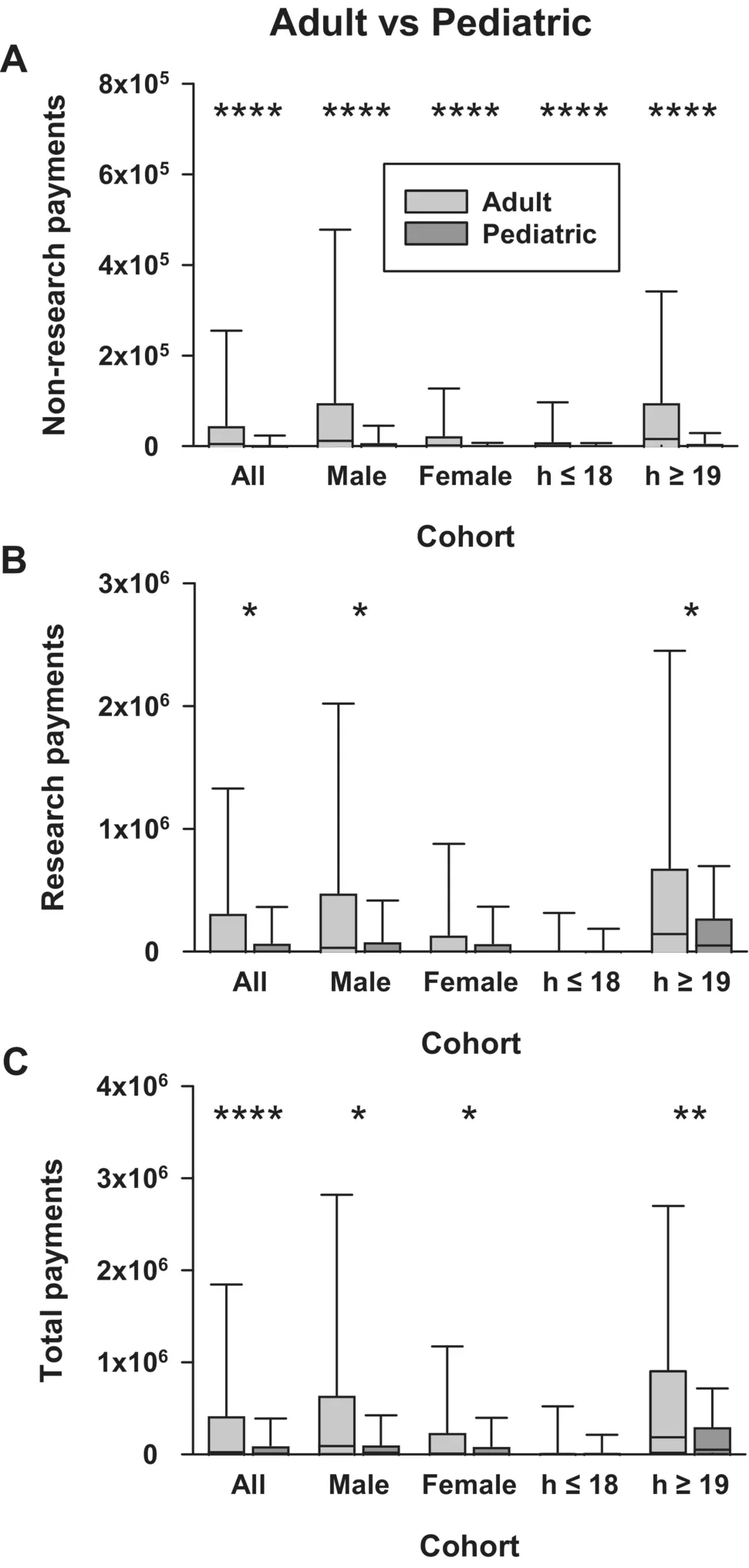
Industry payments to PRAs and ARAs. (A–C) Results are presented as box plots of (A) nonresearch‐associated, (B) research‐associated, and (C) total payments to all PRAs (N = 152) and ARAs (N = 567), male PRAs (N = 41) and ARAs (N = 267), female PRAs (N = 111) and ARAs (N = 300), PRAs (N = 95) and ARAs (N = 265) with h ≤18, and PRAs (N = 57) and ARAs (N = 302) with h ≥19. The lines inside the boxes indicate the medians, the outer borders of the boxes indicate the 25th and 75th percentiles, and the bars extending from the boxes indicate the 10th and 90th percentiles. *P ≤ 0.05; **P ≤ 0.01; ****P ≤ 0.001 (adult vs pediatric). ARA, adult rheumatologist–author; h, *h*‐index; PRA, pediatric rheumatologist–author.

**Table 1:** Industry payments to individual US adult and pediatric rheumatologist–authors*

Group	Median, $	25% Percentile, $	75% Percentile, $	Maximum, $	*P* value
Nonresearch associated					
Consulting					**<0.001**
Adult	900	0	25,373	1,284,654	–
Pediatric	0	0	0	921,901	–
Food and beverage					**<0.001**
Adult	434	103	2,048	90,928	–
Pediatric	157	21	440	5,808	–
Travel and lodging					**<0.001**
Adult	0	0	3,530	446,606	–
Pediatric	0	0	0	54,358	–
Honoraria					**<0.001**
Adult	0	0	0	693,473	–
Pediatric	0	0	0	24,625	–
Ownership					0.608
Adult	0	0	0	5,588,117	–
Pediatric	0	0	0	13,644	–
Other					**<0.001**
Adult	20	0	3,536	228,800,232	–
Pediatric	0	0	48	126,000	–
Total nonresearch					**<0.001**
Adult	4,905	213	46,465	230,330,846	–
Pediatric	244	46	3,830	1,087,794	–
Research associated					
Research payments					**0.008**
Adult	0	0	41	469,945	–
Pediatric	0	0	0	34,403	–
Associated funding					**0.020**
Adult	0	0	306,256	25,053,949	–
Pediatric	0	0	67,783	5,435,531	–
Total research					**0.018**
Adult	270	0	314,030	25,209,872	–
Pediatric	0	0	70,324	5,457,351	–
Total payments					**<0.001**
Adult	21,030	516	419,086	230,358,581	–
Pediatric	5,744	181	95,310	6,545,144	–

* Results are presented as industry payments rounded to the nearest dollar. There were 567 adult and 152 pediatric rheumatologist–authors in each category. *P* values ≤0.05 are bolded.

### Industry payments to PRAs versus ARAs by sex

The sex distribution of the PRAs differed from that of the ARAs, with 73.0% (111 of 152) of the PRAs versus 52.9% (300 of 567) of the ARAs being female (*P* < 0.001). Because industry payments are greater to male than to female rheumatologist–authors (when PRAs and ARAs are lumped together),^10^, ^11^ we separately analyzed male PRAs versus ARAs and female PRAs versus ARAs. Nonresearch‐associated, research‐associated, and total payments were lower to the 41 male PRAs than to the 267 male ARAs and were lower to the 111 female PRAs than to the 300 female ARAs (Figure 1A–C).

### Relation of industry payments to *h*‐index

In addition to the association between industry payments to US physician–authors and the recipient's sex, industry payments correlate with author impact as measured by the *h*‐index.^11^ To assess whether the lower industry payments to PRAs than to ARAs were related to differences in *h*‐indexes, we used the median *h*‐index of 18 for the 719 US rheumatologist–authors to divide the PRAs and ARAs into low *h*‐index (≤18) and high *h*‐index (≥19) groups. Nonresearch‐associated, research‐associated, and total payments were lower to the high *h*‐index PRAs than to the high *h*‐index ARAs and were lower to the low *h*‐index PRAs than to the low *h*‐index ARAs (Figure 1A–C). Moreover, these disparities persisted when both author sex and author *h*‐index were considered. Although there were no significant differences in *h*‐indexes between male PRAs and ARAs or between female PRAs and ARAs (*P* ≥ 0.101; Figure 2A), payments were lower to the PRAs than to the ARAs (Figure 2B–D).

**Figure 2:**
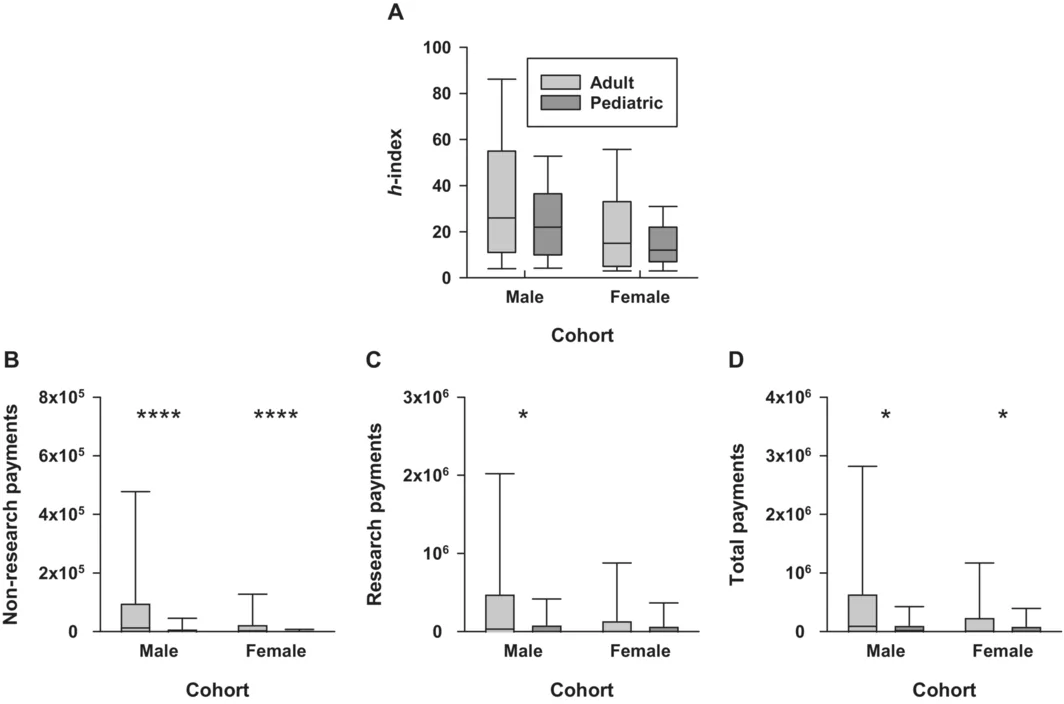
Relation of industry payments to *h*‐index. (A) Results are presented as box plots of the *h*‐indexes of the male and female PRAs and ARAs as in Figure 1. (B–D) Results are presented as box plots of (B) Non‐res, (C) Res, and (D) total payments to the 41 male PRAs, 267 male ARAs, 111 female PRAs, and 300 female ARAs. *P ≤ 0.05; ****P ≤ 0.001 (adult vs pediatric). ARA, adult rheumatologist–author; Non‐res, nonresearch associated; PRA, pediatric rheumatologist–author; Res, research associated.

### High‐end research‐associated industry payments to PRAs versus ARAs

Whereas any research‐associated funds are welcome to academicians and their academic programs, the large ones have the greatest impact. Of the 152 PRAs who received industry payments, 70 (46.1%) received research‐associated payments, and of the 567 ARAs who received industry payments, 288 (50.8%) received research‐associated payments (*P* = 0.299). Remarkably, the magnitude of research‐associated payments during the vetted seven‐year period was ≥$100,000 for 195 (67.7%) of these ARAs but for only 33 (47.1%) of these PRAs (*P* = 0.001) and was ≥$1,000,000 for 71 (24.7%) of these ARAs but for only 3 (4.3%) of these PRAs (*P* < 0.001). Moreover, five of these ARAs but none of these PRAs received ≥$10,000,000 in research‐associated payments.

### Relationship between research‐associated and nonresearch‐associated industry payments to PRAs versus ARAs

Nonresearch‐associated, research‐associated, and total payments were lower to the 70 PRA recipients of research‐associated payments than to the corresponding 288 ARA recipients (Figure 3A). As with the parent cohorts, significantly lower payments to the PRAs than to the ARAs were observed for all OPD payment categories other than ownership (Table 2). Importantly, nonresearch‐associated payments correlated significantly with research‐associated payments among the PRAs (r = 0.325, *P* = 0.006) and the ARAs (r = 0.456, *P* < 0.001), with the regression line for the PRAs being at least an order of magnitude below that for the ARAs (Figure 3B). Similar relationships were observed when male PRAs were compared to male ARAs (Figure 3C) and when female PRAs were compared to female ARAs (Figure 3D). That is, at any given level of research‐associated payment, the corresponding nonresearch‐associated payment to a PRA was substantially lower than that to an ARA. Overall, the aggregate nonresearch‐associated payments were $1,728,610 (mean $24,694 per recipient) to these 70 PRAs compared to $295,155,467 (mean $1,024,845 per recipient) to the corresponding 288 ARAs.

**Figure 3:**
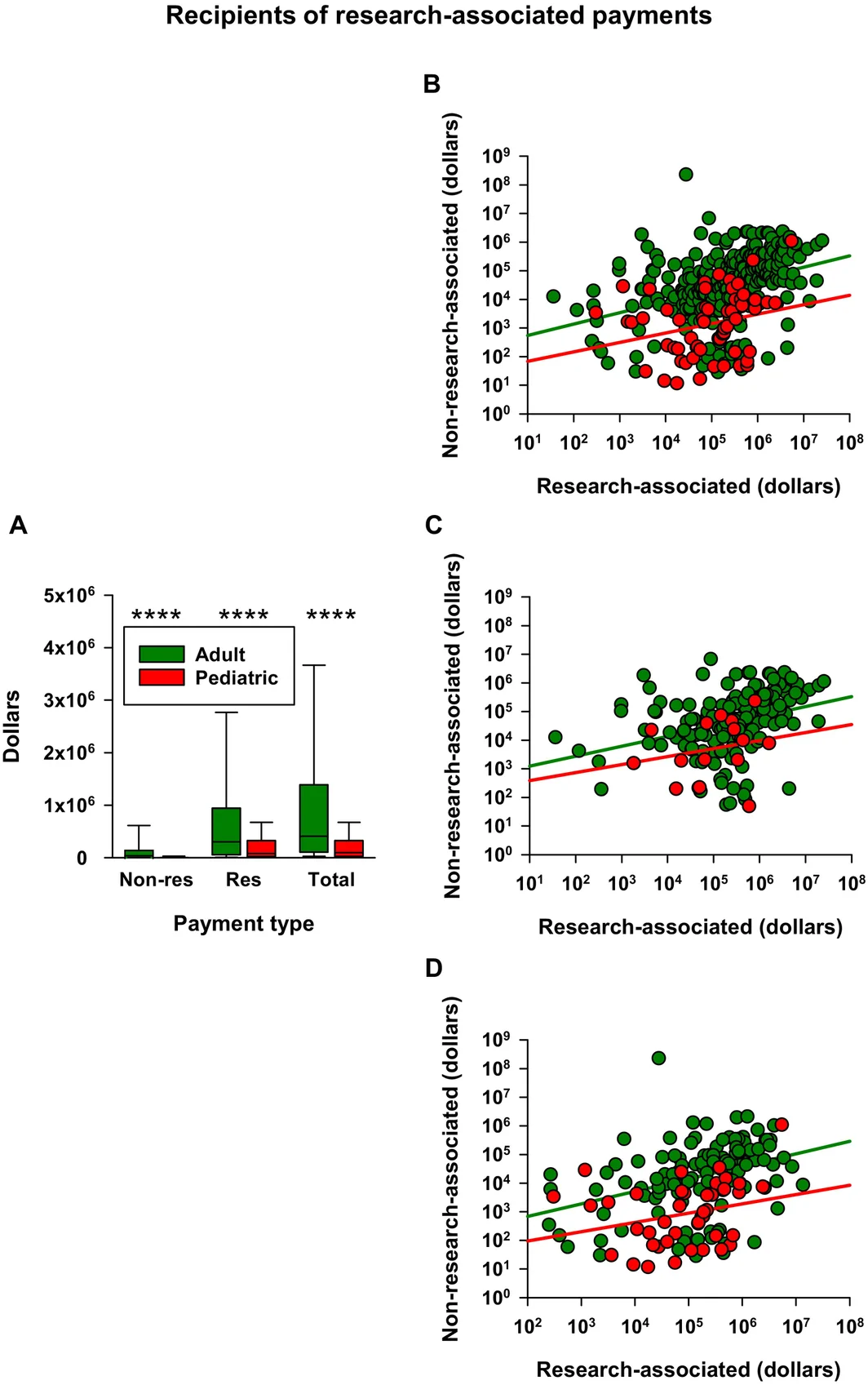
Research‐associated payments to PRAs and ARAs and their correlations with nonresearch‐associated payments. (A) Results for nonresearch‐associated payments, research‐associated payments, and total payments are presented as in Figure 1 for the 70 PRAs and 288 ARAs who received research‐associated industry payments during the 2017 to 2023 interval. (B–D) The scatter plots indicate nonresearch‐associated industry payments versus research‐associated industry payments in dollars to (B) all 70 PRAs (red) and 288 ARAs (green), (C) the 20 male PRAs (red) and 160 male ARAs (green), and (D) the 50 female PRAs (red) and128 female ARAs (green) who received research‐associated payments. The red and green solid lines indicate the respective linear regression lines. Because the Y axis is plotted on a log scale, the circles associated with no (zero) nonresearch‐associated payments are not seen graphically but contribute to the calculation of the regression lines. ****P ≤ 0.001. ARA, adult rheumatologist–author; PRA, pediatric rheumatologist–author.

**Table 2:** Industry payments to individual US adult and pediatric rheumatologist–authors who received research‐associated payments*

Group	Median, $	25% Percentile, $	75% Percentile, $	Maximum, $	*P* value
Nonresearch associated					
Consulting					**<0.001**
Adult	18,307	2,441	89,245	1,284,654	**–**
Pediatric	0	0	1,919	921,901	–
Food and beverage					**<0.001**
Adult	1,002	229	3,629	90,928	**–**
Pediatric	145	0	456	5,118	–
Travel and lodging					**<0.001**
Adult	2,291	0	19,407	446,606	**–**
Pediatric	0	0	9	54,358	–
Honoraria					**<0.001**
Adult	0	0	4,755	693,473	**–**
Pediatric	0	0	0	24,625	–
Ownership					0.518
Adult	0	0	0	5,588,117	–
Pediatric	0	0	0	13,644	–
Other					**<0.001**
Adult	454	0	20,347	228,800,232	**–**
Pediatric	0	0	950	88,709	–
Total nonresearch					**<0.001**
Adult	37,040	5,863	162,358	230,330,846	**–**
Pediatric	532	27	5,400	1,087,794	–
Research‐associated					
Research payments					**0.007**
Adult	18	0	7,699	469,945	**–**
Pediatric	0	0	2,519	34,403	–
Associated funding					**<0.001**
Adult	286,244	51,978	938,532	25,053,949	**–**
Pediatric	77,706	19,743	324,179	5,435,531	–
Total research					**<0.001**
Adult	301,461	55,125	942,359	25,209,872	**–**
Pediatric	79,250	19,743	324,179	5,457,351	–
Total payments					**<0.001**
Adult	410,138	105,671	1,385,846	230,358,581	**–**
Pediatric	97,719	21,929	326,010	6,545,144	–

* Results are presented as industry payments rounded to the nearest dollar. There were 288 adult and 70 pediatric rheumatologist–authors in each category. *P* values ≤0.05 are bolded.

## DISCUSSION

Several observations emerge from our study. First, acceptance of industry payments among US PRAs and ARAs is widespread, with more than three out of every four of each having received at least one industry payment during the vetted seven‐year period. For industry, the money may be well spent because industry funding has been associated with positive outcomes in reported drug trials. Of 600 clinical trials published in 2019 or later that were highly cited through December 2022, 409 were industry funded, and 364 (89.0%) of these reached conclusions favoring the sponsor, with 90 of 92 (97.8%) of the nonrandomized trials favoring the sponsor.^13^ Moreover, industry payments have been associated with increased prescriptions and Medicare spending for the related drugs,^9^ thereby adding to the industry “bottom line.”

Although industry payments raise concerns of conflicts of interest,^8^, ^9^ they, nonetheless, offer tangible benefits/value for practitioners and for the public. Medical and scientific investigation is costly, and breakthrough investigations remain largely dependent on extramural financial support by government, foundations, philanthropists, and industry. Industry payments may provide the support needed to execute important studies not funded by government agencies or charitable foundations, and industry payments to physicians may serve as a financial lifeline for struggling gifted investigators. Reassuringly, previous comparisons between industry‐funded and nonindustry‐funded randomized controlled trials (RCTs) in systemic lupus erythematosus (SLE) and rheumatoid arthritis failed to show any publication bias,^14^, ^15^ suggesting that publications arising from industry support may have no more impact on the readership than do publications not supported by industry. Although the lay public remains largely unaware that industry payments to physicians are publicly available,^16^, ^17^ those aware that their physicians accept industry payments often display decreased trust in those physicians,^18^, ^19^ with the caveat that trust is not affected when the physician himself apprises the patient of a financial relationship with industry.^20^

Our second observation is that a meaningful minority (152 of 719 [21.1%]) of the US rheumatologist–authors who received industry payments are PRs. Although the precise numbers of US ARs and PRs are not known, the number of “clinically active” ARs in 2019 was 5,667, as determined through Medicare claims;^21^ the number of PR full‐time equivalents was 287, as determined through a workforce model conducted by the ACR in 2015; and the American Board of Pediatrics counted 479 US pediatricians, age 70 years and younger, as certified PRs in 2024.^22^ If the true ratio of US ARs to US PRs is approximately 11.8 (5,667:479) or approximately 19.7 (5,667:287), then the 3.7 ratio of ARAs to PRAs who received industry payments (567:152) suggests that PRA recipients of industry payments are not underrepresented.

This non‐underrepresentation notwithstanding, our third and highly jarring observation is that the magnitude of payments to the PRAs was markedly lower than that to the ARAs. When considering the 567 ARAs and 152 PRAs who received any industry payments, median total, nonresearch‐associated, and research‐associated payments to the PRAs were 27.3% ($5,744 vs $21,030), 5.0% ($244 vs $4,905), and 0.0% ($0 vs $270) of those to the ARAs. In general, lifetime earnings for academic pediatric subspecialists, including PRs, are substantially lower than those for their adult counterparts.^23^ One can speculate that part of the disparity in rheumatology stems from the disparity in research‐associated support that industry affords ARs versus PRs. An academic AR who procures substantial research‐associated industry funds for his or her academic division would almost certainly receive greater compensation than an academic PR who procures lesser amounts for his or her division.

When considering just the 288 ARAs and 70 PRAs who received research‐associated payments, median total, nonresearch‐associated, and research‐associated payments to the PRAs were 23.8% ($97,719 vs $410,138), 1.4% ($532 vs $37,040), and 26.3% ($79,250 vs $301,461) of those to the ARAs. Moreover, high‐end research‐associated payments were overwhelmingly skewed away from the PRAs to the ARAs. Aggregate total, nonresearch‐associated, and research‐associated payments to the PRAs were 3.9% ($24,214,466 vs $620,617,911), 0.8% ($2,345,683 vs $298,369,856), and 6.8% ($21,868,783 vs $322,248,055) of those to the ARAs. These gaps between PRAs and ARAs cannot simply be explained by differences in sex distribution because payments to male and female PRAs were each lower than those to the corresponding ARAs. Furthermore, these gaps cannot be explained by differences in author impact because payments to high *h*‐index and low *h*‐index PRAs were each lower than those to the corresponding ARAs. Because there are far fewer children than adults who suffer from rheumatic disease, the market for therapeutics and diagnostics for pediatric rheumatic disease is less lucrative than the corresponding market for adult rheumatic disease. Moreover, there are more regulatory hurdles associated with pediatric studies than with adult studies, so it should be expected that the sum of industry payments to all ARs (and ARAs) would be greater than the sum of industry payments to all PRs (and PRAs). However, payments per recipient should not necessarily differ, so the substantially lower industry payments per recipient to PRAs than to ARAs are unsettling.

Our fourth and equally jarring observation focuses on the PRAs and ARAs who received research‐associated payments. The magnitude of nonresearch‐associated payments to the PRAs was considerably lower than that to the ARs at any level of research‐associated payment, with aggregate nonresearch‐associated payments to the PRA recipients of research funds being 0.6% ($1,729,610 vs $295,155,467) of those to the corresponding ARA recipients. That is, among authors who were actively engaged in industry‐sponsored research endeavors, nonresearch‐associated payments were markedly lower to the PRAs than to the ARAs.

On a practical level, our findings suggest that both research funding and income supplementation from industry sources are less available to PRs than to ARs. For many pediatric subspecialties, including rheumatology, anticipated lifetime earnings for the subspecialist is lower than that for the general pediatrician.^24^ This reinforces the notion that pediatric rheumatology and PRs need additional financial support, especially in light of the “endangered” status of the physician–scientist in pediatric rheumatology.^25^ The negative financial impact of fellowship training in pediatric rheumatology likely dissuades a percentage of graduating pediatric residents from applying to pediatric rheumatology training programs and may well contribute to the shortfall of PRs in the United States,^6^ Canada,^26^ Australia and New Zealand,^27^ and Latin America.^28^

The cause of the wide support gap between PRs and ARs is not immediately apparent. Regarding research‐associated payments, simple economics may be the driving factor because the number of children who suffer from rheumatic disease is far smaller than the number of adults who suffer from rheumatic disease. A more charitable possibility is that fewer pediatric providers and pediatric patients are available for recruitment to industry‐sponsored initiatives. For example, initial approval by the US Food and Drug Administration (FDA) of belimumab for adults with SLE followed a phase 1 RCT with 70 patients with SLE,^29^ a phase 2 RCT with 449 patients with SLE,^30^ and two phase 3 RCTs with 867 and 819 patients with SLE, respectively.^31^, ^32^ In contrast to these 2,205 adult patients with SLE, FDA approval for belimumab for childhood SLE followed a single phase 2 trial with a mere 93 patients with SLE.^33^ Regarding the gap in nonresearch‐associated payments, economics may again play a role in that industry may not sponsor “education” programs or offer consulting opportunities geared to pediatric rheumatic disease to the same extent as sponsored and offered for adult rheumatic disease. Alternatively, and not mutually exclusively, academic pediatric providers may be more institutionally restricted from, or more culturally averse to, accepting industry payments than their adult colleagues.

The disparity between PRs and ARs may also relate to an evolving landscape in the US rheumatology workforce. Whereas recognition of adult rheumatology as a distinct specialty can be traced to the 1940s, pediatric rheumatology was not recognized as a distinct subspecialty by the American Board of Pediatrics until 1992.^34^ Accordingly, there are more veteran ARs than veteran PRs. Given that name recognition is alluring to industry, industry will likely preferentially enlist veteran individuals with well‐established track records over less‐established ones, leading to greater engagement (and payments) to the collectively more veteran ARAs than to the collectively less veteran PRAs. As pediatric rheumatology continues to “mature” and PRs continue to build their reputations, industry support of PRs should (and, we submit, must) increase.

One can wonder whether underpayment by industry to pediatric versus adult investigators extends beyond rheumatology. During the 2022 to 2026 interval, the number of applicants per available position ranged from 1.27 to 1.36 for adult rheumatology but only from 0.57 to 0.74 for pediatric rheumatology. This striking disparity was not limited to rheumatology but extended to several, but not all, disciplines as well (https://www.nrmp.org/match‐data/2025/02/results‐and‐data‐specialties‐matching‐service‐2026‐appointment‐year/). Studies comparing industry payments to adult versus pediatric subspecialists across multiple disciplines are warranted.

There are limitations to the present study. First, our results are contingent on accurate reporting of data to the OPD. Nonetheless, it is highly unlikely that chance inaccuracies would systematically lead to significant differences between PRAs and ARAs. Second, we limited our analysis to the three ACR journals. Although there certainly are publications of great interest to rheumatologists in other journals, the ACR journals serve as major “go‐to” journals for both adult and pediatric US rheumatologists. Third, we could not determine how much of the differences in payments were due to differences in invitation versus acceptance rates among prospective PR and AR participants in industry activities.

In summary, although both PRAs and ARAs are common recipients of industry payments, the magnitude of payments per recipient is far lower to the former than to the latter. This disparity cannot readily be explained by differences in sex distribution or differences in author impact. We call upon industry to redouble its efforts in support of PRs and pediatric rheumatology. We also call upon academic centers to revisit their policies and attitudes regarding receipt by their faculties of industry funding. We acknowledge that these recommendations are controversial, but they are neither hateful nor prejudicial. The workforce crisis facing pediatric rheumatology is real, and proposed fixes to date either have not been implemented or have simply not worked. Industry support to US PRAs and ARAs is already highly prevalent, and we collectively have been able to successfully handle the attendant downsides. Accordingly, we need to think “outside the box” and not dismiss out of hand any possible remedies, including those with manageable drawbacks.

## AUTHOR CONTRIBUTIONS

All authors contributed to at least one of the following manuscript preparation roles: conceptualization AND/OR methodology, software, investigation, formal analysis, data curation, visualization, and validation AND drafting or reviewing/editing the final draft. As corresponding author, Dr Stohl confirms that all authors have provided the final approval of the version to be published and takes responsibility for the affirmations regarding article submission (eg, not under consideration by another journal), the integrity of the data presented, and the statements regarding compliance with institutional review board/Declaration of Helsinki requirements.

## Supporting information


**Disclosure Form**:
